# Growing and evolving: remarks for the 35^th^ anniversary of the founding of *Zoological Research*

**Published:** 2015-01-18

The beginning of a new year is always a time for reflection, contemplation and optimism. For the past thirty-five years, since its founding in 1980, *Zoological Research* (*ZR*) has experienced many highs and lows, seeing great advances but also overcoming numerous hurdles. *Zoological Research* has made many valuable contributions to the progress of biological sciences in China and won prestige both at home and abroad. Currently, it serves as a key journal focusing on Genome Evolution and Genetic Diversity, Animal Ecology and Ethology, Primates and Animal Models of Human Diseases. The journal also publishes high quality papers related to taxonomy and DNA barcoding, developmental biology, physiology, biochemistry, immunology, and neuroscience. The growth achieved by *ZR* is not only due to our excellent readers and reviewers, but also to the evolution of our authors into renowned scholars. Accordingly, we would like to thank you all for your constant support and generous help.

*Zoological Research* has always put the needs of its readers and authors first. To facilitate communication between our domestic colleagues and the international community, in 2013 to 2014 we successfully transitioned from a Chinese-language only journal, into a Chinese-English bilingual journal and finally into an internationally aimed English-language only journal. To help expand author view-points, we also included new Editorial and Letter to the Editor columns. Furthermore, the editorial board and editorial members continued to refine our publishing period, editing quality, typesetting, and journal marketing. In addition to *ZR* being selected as a high-quality publication for the dissemination of research findings, both the visibility and influence of *ZR* has continued to increase.

As a respectable scientific publication in China, *ZR* had made impressive progress over the past few years, particularly in regards to its high academic value and leading role in advancing the quality of academic journals in China. In 2014, *ZR* was ranked among the top 300 “Outstanding S&T Journals of China”, the second time* ZR* has been recognized by this prestigious award since 2008. In addition, four articles published in *ZR* between 2009 to 2013 were also included in the “Project of Frontrunner 5000” (F5000), which aims to promote scientific communication and the internationalization of Chinese S&T journals.

The contributions from our colleagues have enabled our continued growth and advance. From a pool of more than 4600 journals, *ZR* was among the top 10% of Chinese publications and was awarded the “The Highest / The International Impact Academic Journals of China” in Science, Technology and Engineering from 2012 to 2014 for its achievements in internationalizing life science research, as determined by its increasing international annual citation frequency and international impact factor.

In September 2014, we were greatly honored to have a group of renowned academic experts from home and abroad join us as members of the editorial board. Thus, with dedicated assistance from our editorial team and first-rate reviewers, we will continue to expand and improve our publication, and in particular we welcome manuscripts on Genetics and Evolution, Primates and Animal Models of Human Diseases, and Biodiversity Conservation and Ecology. We also extend a special invitation to our colleagues to serve as single-issue Guest Editors, and if any author would like to advance a topic that is prospective, controversial and worth exploring, please feel free to contact us.

Once again, we would like to thank you all for your enduring support and faith. Our continued growth and evolution together will ensure that *ZR* remains a respected publication platform that provides ever greater possibilities to you.

Sincerely yours,


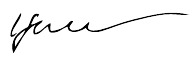


Yong-Gang YAO, Editor-in-Chief

*Kunming Institute of Zoology*, *Chinese Academy of Sciences*, *Kunming *650223, *China*


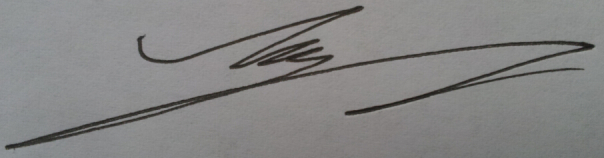


Yun ZHANG, Executive Editor-in-Chief

*Kunming Institute of Zoology*, *Chinese Academy of Sciences*, *Kunming *650223, *China*

